# Effect of tocopherol supplementation during last trimester of pregnancy on mRNA abundances of interleukins and angiogenesis in ovine placenta and uterus

**DOI:** 10.1186/1477-7827-10-4

**Published:** 2012-01-23

**Authors:** Ramanathan K Kasimanickam, Vanmathy R Kasimanickam, Gary J Haldorson, Ahmed Tibary

**Affiliations:** 1 College of Veterinary Medicine, Washington State University, Pullman, WA 99164, USA

## Abstract

**Background:**

Interleukins (IL) play an important role in angiogenesis. Tocopherol possesses immunomodulating effect in addition to antioxidant property. The objective of this study was to determine whether gamma tocopherol's (gT) angiogenic activity in placental network is enhanced via promoting interleukins.

**Methods:**

Pregnant ewes (N = 18) were supplemented, orally, with 500 mg of alpha tocopherol (aT; N = 6) or 1,000 mg of gT (N = 7) or placebo (CON; N = 5) once daily from 107 to 137 days post breeding. Uterine and placental tissue samples were obtained at the end of supplementation to evaluate relative mRNA expressions of IL-1b, IL-6, IL-8, Tumor Necrosis Factor (TNF) alpha, Vascular Endothelial Growth Factor (VEGF), kinase insert domain receptor (KDR; VGFR2; a type III receptor tyrosine kinase), and soluble fms-like tyrosine kniase-1 (sFlt1 or sVEGFR1) in uterus, caruncle and cotyledon.

**Results:**

Oral supplementation of gT increased IL-6, IL-8, KDR and VEGF mRNA abundances whereas sFlt1 mRNA abundance was suppressed in uterus, caruncle and cotyledon, compared to aT and placebo treated ewes (*P *< 0.05). The TNF alpha and IL-1b mRNA abundances were suppressed in uterus, caruncle and cotyledon but TNF alpha is higher in gT group compared to aT group (*P *< 0.05), whereas IL-1b was similar between treatment groups (*P *> 0.1).

**Conclusions:**

Gamma tocopherol supplementation increased IL-6, IL-8, and KDR mRNA abundances and suppressed sFlt1 and TNFalpha mRNA abundances thereby increased VEGF mRNA expression and angiogenesis in placental vascular network during late gestation. It is plausible that the angiogenic effect of gamma tocopherol in placental vascular network is exerted via an alternate path by enhancing IL-6 and IL-8.

## Background

Angiogenesis refers to the formation of new vascular beds, and is a critical process for normal placental growth and development [1-3]. Although numerous factors have been implicated in angiogenesis, recent observations have led to the identification of the major factors regulating the angiogenic process, including those that occur during placental vascularization. These angiogenic factors include the vascular endothelial growth factor (VEGF), fibroblast growth factor (FGF), and the angiopoietin (ANG) protein families, as well as their respective receptors [4-6]. The VEGF is the major angiogenic growth factor of the placenta because this protein probably accounts for most of the angiogenic activity produced by placental tissues [7-11]. The VEGF acts as specific mitogen for endothelial cells through specific membrane receptors, kinase domain-containing receptor (KDR), and the fin-like tyrosine kinase 1(Flt-1) [12]. When VEGF binds to its receptors, it initiates auto-phosphorylation, induces tyrosine kinase activity, and subsequently stimulates cellular responses in normal and abnormal angiogenesis.

Several inflammatory interleukins have been linked with angiogenesis. These interleukins include IL-1, IL-6, and IL-8 [13-28]. Interleukin (IL)-8 acts as a potent chemoattractant for neutrophils, the major cellular component of acute inflammatory infiltrates [13,14]. In addition to this proinflammatory function, there is growing evidence that IL-8 exerts effects on nonimmune cells, including the vascular endothelium [14]. The receptors for IL-8 are widely expressed on normal and various tumor cells and bind IL-8 receptors with high affinity [15]. IL-8 expression correlates with vascularity and microvessel counts [16-18]. Similarly IL-6, a multifunctional cytokine and is a critical factor in various physiological conditions including immune regulation, hematopoiesis and inflammation by modulating a variety of events, such as cell proliferation, differentiation and apoptosis [19-21]. Studies have demonstrated that immunostaining for IL-6 was present in both syncytiotrophoblasts and extra-villous trophoblasts [22]. IL-6 stimulates VEGF synthesis and release, and promotes angiogenesis occurrence in vivo and in vitro [23-26]. These cytokines could play a role in vascular remodeling associated with placentation. The cytokine IL-1 mainly affects inflammatory processes but also possesses various immune, degradative, and growth promoting properties. There are two IL-1 agonistic proteins, IL-1α and IL-1β. Both were shown to contribute to tumor angiogenesis and invasiveness, but the role of IL-1β is more evident in these processes [27].

Tocopherol has immunomodulatory effects, besides its antioxidant properties. Vitamin E is implicated in placental and embryonic development, whereas its deficiency affects embryo survival, and placental and fetal development. Alternative effects of Vitamin E have been recently reported as unrelated to its antioxidant capacity [28]. It induces the expression of VEGF promoter [28]. Vitamin E (15 mg/day) has been able to decrease abortion rate and to increase IL-6 placental levels, while both treatments increased placental levels of VEGF [29]. We have shown that oral supplementation of tocopherol induces angiogenesis in placental vascular network in late pregnant ewes [10]. The hypothesis of the study is that the tocopherol's angiogenic effect following oral supplementation during late gestation in ewes is via promoting interleukins.

The objective of this study is to investigate whether tocopherol's angiogenic activity in placental network is enhanced via promoting interleukins in pregnant ewes during late gestation.

## Methods

### Animals

Eighteen pregnant ewes (2-6 years of age and weighing approximately 68 kg; bred by different sires), with similar breeding dates were selected for the current study. The ewes were maintained under normal pasture conditions until the beginning of the supplementation trial. One week prior to the trial, the selected ewes were moved to the research facility and were penned by treatment groups. The ewes had access to 35 sq ft/ewe paddock lots. In addition they were fed 250 g of concentrate/ewe/day and free choice hay. This study was approved by institutional animal care and use committee at Washington State University (ASAF #03922-001).

### Treatment groups and samples collection

The ewes were randomly assigned to three groups: 1) aT group (n = 6)--received 500 mg of alpha tocopherol (aT; Nature's Bounty, Bohemia, NY 11716), 2) gT group (n = 7)--received 1,000 mg of gamma tocopherol (gT; Kemin Industries Inc., Des Moines, Iowa 50317), 3) Control (CON) group (n = 5)--received a placebo. Animals were supplemented orally, once daily, from approximately 100 to 137 days post breeding (dpb). At the end of the supplementation period (136 ± 1), all ewes were euthanized and tissue samples were collected from the gravid uterus and placentomes to evaluate aT and gT concentrations. Placentomal and uterine tissues were collected close to the umbilical cord for consistency. Tissue samples were snap frozen and stored at -70°C until analysis. Caruncle, cotyledon and intercaruncular uterus samples were also collected and stored in RNAlater (Qiagen Inc., Valencia, CA 91355) and frozen at -70°C to evaluate mRNA expression. Cotyledones were separated from caruncles by applying strong pressure.

### Polymerase chain reaction of selected genes of interest

#### Total RNA extraction from tissues

Total RNA was extracted from uterine and placentomal tissues with RNeasy Mini Kit (QIAGEN Inc. Valencia, CA, USA) according to the manufacturer's protocol. RNA concentration was measured using a NanoDrop spectrophotometer (Thermo Fisher Scientific Inc. West Palm Beach, FL, USA). Sample absorbance ratio of 260/280 wave-length was observed to ensure the purity of RNA and they were close to 2.00. The RNA samples were stored at -20°C until complementary DNA (cDNA) preparation.

### Polymerase chain reaction of selected genes of interest

The mRNA was reverse-transcribed to cDNA. The cDNA samples were prepared using the iScript cDNA Synthesis kit (Bio-Rad, Hercules, CA, USA). A 500 ηg sample of RNA was reverse transcribed in 20 μL reaction at the incubating conditions of 25°C for 5 min, 42°C for 30 min and 85°C for 5 min; 25 ηg/μL RNA equivalent cDNA was obtained. Qiagen Tag PCR master mix (Qiagen, Valencia, CA, USA), a pre-mixed solution was used to amplify the fragment of the genes of interest. Final concentration of the primers was 0.3 μM. Initial denaturation was set at 94°C for 3 min. Followed by 30 cycles of denaturation at 94°C for 1 min, annealing at 55°C for 1 min and extension at 72°C was programmed. A final extension step at 72°C for 10 min was included in thermo-cycling conditions. Primers (Table 1) were designed either using the NCBI website or primer express version 3.0 (Applied Biosystems Inc., Carlsbad, CA, USA). Consideration was given to the set of primers (forward and reverse primers) to ensure separation of at least an intron and melting temperatures and CG content were set at optimal, or close to optimal level. Amplicon was run on a 2% agarose gel and stained with ethidium bromide for visualization to ensure a single amplicon for a set of primers (Additional file 1: Figure S1).

**Table 1:** Primers used for cDNA amplification of the targets by RT-PCR

Gene	Primer	Seq 5' to 3'	Product length	Accession number
IL-1b	Forward	TCACAGGAAATGAGCCGAGAA	150	NM_001009465

	Reverse	CAGCTGCAGGGTCGGTGTAT		

IL-6	Forward	ACACCACCCCAAGCAGACTACT	200	NM_001009392

	Reverse	CCCAGATTGGAAGCATCCAT		

IL-8	Forward	GCCAGAAGAAACCTGACAAAAAG	220	NM_001009401

	Reverse	GCAGTGTGGCCCACTCTCA		

VEGF	Forward	CCTCACCAAAGCCAGCACAT	150	AF071015.1

	Reverse	CGTCTGCGGATCTTGTACAAAC		

KDR	Forward	GATGCTCGCCTCCCTTTGA	250	AF233076

	Reverse	GATCCCCATGCCAGCAATC		

SFlt1	Forward	GCCACGCCTGAAATCTACCA	150	AF233077

	Reverse	GGCGTTGAGCGGAATGTAGT		

TNFα	Forward	GACCCTCCTCATCCCCTTCT	300	NM_001024860.1

	Reverse	AGCCCACCCATGTCAAGTTC		

β-actin	Forward	CCAAGGCCAACCGTGAGA	86	NM_001009784.1

	Reverse	AGCCTGGATGGCCACGT		

Ovine Interleukin (IL)-1b, IL-6, IL-8, Vascular Endothelial Growth Factor (VEGF), Kinase insert Domain Receptor (KDR), soluble Fms-Like Tyrosine kniase-1 (sFlt1), Tumor Necrosis Factor (TNF) α and β-actin cDNA were amplified by RT-PCR from the uterus and placenta total RNA using the above set of primers

### Determination of mRNA expression using real-time PCR

SYBR green chemistry was applied to observe relative mRNA expression. Fast SYBR green master mix (2×) (Applied Biosystems Inc., Carlsbad, CA, USA) was used to prepare the reaction mix. The final concentration of each primer was 0.3 μM. A 20 μL aliquot of three technical replicates were used for each sample. A 1.6 μL volume of 25 ng/μL RNA equivalent cDNA was present in the total volume of the three triplicates. StepOne Plus instrument (Applied Biosystems Inc., Carlsbad, CA, USA) was used for the real time PCR runs. Pre-cycling stage was maintained at 95°C for 20 s. Forty cycle amplification was carried out with the conditions of 95°C for 3 s and 60°C for 30 s (fast ramp speed conditions for the fast mixture). A continuous dissociation step was added to look for additional amplification products.

Carboxy X rhodamine (ROX) dye was set up for the passive internal reference. The baseline was automatically adjusted to obtain threshold cycles of each sample. Threshold cycles were normalized to an endogenous control, β-actin. A standard curve was obtained using 1 in 5 dilutions for each set of primer in order to check the amplification efficiency. Correlation co-efficient for the dilution curve was ≥ 0.9900.

### Statistical analysis

The RT-PCR data were subjected to ANOVA (SAS Version 9.12, Cary, NC, USA) using 2^-ΔΔCt ^values to ascertain statistical significance of any differences in IL-1b, IL-6, IL-8, TNFα, VEGF, KDR, and sFlt1 expression in placebo vs. tocopherol-treated groups in the placenta and uterus [10,30]. There were 3 ewes (2 in CON and 1 in aT groups) with twins. As the outcome remained the same whether or not the values from the second fetus of twins were included in the analysis, the results presented here included values from one fetus of the twins. It was hypothesized that the mean differences in mRNA expressions will be 7 fold differences in treated compared to control groups. To detect the same differences in the mean mRNA expression, with adequate statistical power (1-β = 0.8) and statistical significance (α = 0.05), the study will need a sample size of 3 ewes per treatment group.

## Results

**Figure 1:**
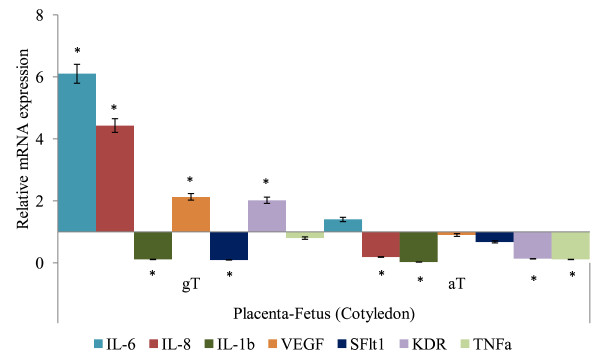
**Relative mRNA of target genes expressed in cotyledon of late pregnant ewes following oral supplementation of alpha tocopherol, gamma tocopherol or placebo**. a. Interleukin(IL)-6, IL-8, IL-1b, Vascular Endothelial Growth Factor (VEGF), Kinase insert Domain Receptor (KDR), soluble Fms-Like Tyrosine kniase-1 (sFlt1) and Tumor Necrosis Factor (TNF) α mRNA expressions in cotyledon following gamma tocopherol (gT) or alpha tocopherol (aT) oral supplementation (× expression relative to control = 1; * *P *< 0.05).

**Figure 2:**
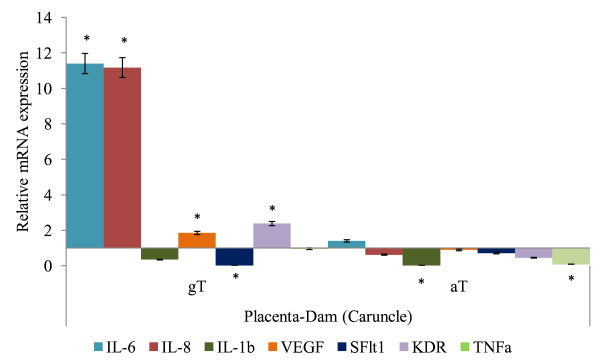
**Relative mRNA of target genes expressed in caruncle of late pregnant ewes following oral supplementation of alpha tocopherol, gamma tocopherol or placebo**. a. Interleukin(IL)-6, IL-8, IL-1b, Vascular Endothelial Growth Factor (VEGF), Kinase insert Domain Receptor (KDR), soluble Fms-Like Tyrosine kniase-1 (sFlt1) and Tumor Necrosis Factor (TNF) α mRNA expressions in caruncle following gamma tocopherol (gT) or alpha tocopherol (aT) oral supplementation (× expression relative to control = 1; * *P *< 0.05).

**Figure 3:**
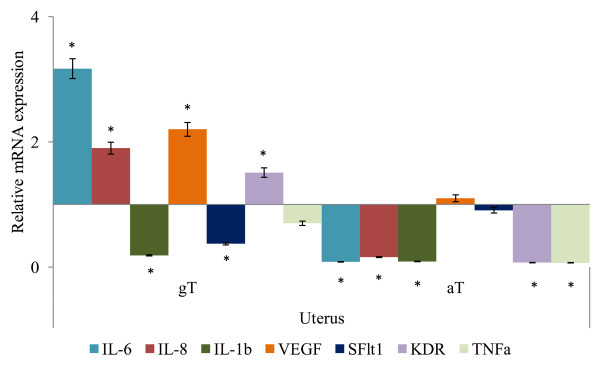
**Relative mRNA of target genes expressed in uterus of late pregnant ewes following oral supplementation of alpha tocopherol, gamma tocopherol or placebo**. Interleukin(IL)-6, IL-8, IL-1b, Vascular Endothelial Growth Factor (VEGF), Kinase insert Domain Receptor (KDR), soluble Fms-Like Tyrosine kniase-1 (sFlt1) and Tumor Necrosis Factor (TNF) α mRNA expressions in uterus following gamma or alpha tocopherol oral supplementation (× expression relative to control = 1; * *P *< 0.05).

The mRNA expressions of IL-6, IL-8, IL-1b, TNF-α, VEGF, sFlt1 and KDR in placenta and uterus in tocopherol supplemented groups relative to CON (where CON group is 1), are given in Figures 1, 2 and 3. In cotyledon (Figure 1) and caruncle (Figure 2), the IL-6, IL-8, VEGF and KDR in ewes supplemented with gT were significantly higher in abundance compared to the CON group (*P *< 0.05). The IL-1b, sFlt1 mRNA abundances in cotyledon (Figure 1) and sFlt1 mRNA abundance in caruncle (Figure 2) were lower (*P *< 0.05) in gT group compared to the CON groups. The supplementation of aT suppressed mRNA expression of IL-8, IL-1b, TNF-α and KDR in cotyledon (Figure 1), and IL-1b and TNF-α in caruncle (Figure 2) compared to placebo treated ewes (*P *< 0.05).

In the uterus, the IL-6 and IL-8, VEGF and KDR mRNA expressions in ewes supplemented with gT were significantly higher in abundance, where as IL-1b and sFlt1 was significantly lower in abundance compared to the CON group (Figure 3). The IL-6, IL-8, IL-1b and KDR and TNF-α mRNA abundance in ewes supplemented with aT were significantly lower compared to the CON group (Figure 3).

Oral supplementation of gT increased IL-6, IL-8, KDR and VEGF mRNA expression whereas sFlt1 mRNA expression was suppressed in cotyledon (Figure 1), caruncle (Figure 2) and uterus (Figure 3) compared to aT treated ewes (*P *< 0.05). The mRNA abundances of both TNF-α and IL-1b was suppressed in uterus, caruncle and cotyledon but TNF-α was different between gT and aT treatment groups whereas IL-1b was similar between treatment groups (*P *> 0.1).

## Discussion

In this study we observed that supplementation of gT to pregnant ewes during late gestation increased IL-6, IL-8, KDR and VEGF mRNA abundances in uterus, caruncle and cotyledon whereas the mRNA expression of IL-1b, sFlt1 was suppressed compared to placebo treated ewes. In contrast, supplementation of aT suppressed mRNA expression of IL-1b, IL-6 and KDR in both cotyledon and caruncle, and IL-8 in cotyledon, compared to placebo treated ewes (*P *< 0.05).

In our previous study, we showed that the tocopherol concentrations of uterus and placentome increased following oral supplementation of tocopherol during late gestation in pregnant ewes [10]. IL-8 signaling has been shown to promote the transactivation of the epidermal growth factor receptor in vascular endothelial cells [31,32], promoting downstream activation of MAPK signaling. In addition, IL-8 signaling has been shown to induce the phosphorylation of the KDR, (VEGFR-2) in endothelial cells, regulating the permeability of the endothelial barrier [33]. Protein tyrosine kinases are a further downstream target of IL-8 signaling responses in endothelial cells [31]. In our previous study, we demonstrated increased fractal dimension and lowered lacunarity, signs for improved angiogenesis, in placental vascular network following gT supplementation compared to aT supplemented and control ewes during late gestation [10]. In this study, we demonstrated increased IL-8, KDR and VEGF mRNA abundances in gT supplemented ewes. The stimulatory effect of IL-8 might plausibly be regulated by increased VEGF mRNA expression, which is enhanced via both VEGFR1 (low sFlt1 mRNA abundance) and VEGFR2 (high KDR mRNA abundance).

Fan et al. (2008) showed that interleukin (IL)-6 increased endothelial progenitor cell proliferation, migration and in vitro angiogenic-like tubulogenesis [22]. It appears that lower IL-6 levels may induce a deregulation leading to an exacerbated inflammation, a poor angiogenesis and fetal death by ischemia [29]. However, it should be noted that the underlying angiogenic mechanism is that IL-6 upregulates KDR. IL-6 triggers VEGF-induced angiogenic activity through increasing VEGF release, up-regulates KDR expression and phosphorylation through activating ERK1/2 signaling, and stimulates MMP-9 overexpression [34,35]. It is evident, in this study, that ewes supplemented with gT had increased IL-6, VEGF and KDR causing enhanced angiogenesis in the placental vascular network in that group.

Numerous studies demonstrated a role for IL-6 in increased blood pressure. For example, plasma levels of IL-6 are strongly associated with hypertension in humans and can be reduced by administration of Ang II receptor antagonists [36-38]. Animal studies showed that infusion of IL-6 induces hypertension in pregnant rats [39,40]. Trophoblast cells are capable of responding to IL-6 [41-43]. Trophoblast cells have a JAK/STAT-signaling pathway that functions downstream of IL-6R activation. JAK/STAT signaling, downstream of IFN-γ stimulation, is responsible for increased sFlt-1 production by human corneal fibroblasts [44]. The authors speculated that the IL-6 stimulates sFlt-1 and sEng production by trophoblast cells in an autocrine manner by JAK-STAT signaling, and that increased IL-6 contributes to antibody-mediated hypertension in women with PE via IL-6-induced stimulation of sFlt-1 production by trophoblasts [44]. Interestingly in this study, sFlt-1 mRNA expression is suppressed in tocopherol treated groups plausibly explaining that ischemic necrosis is prevented by such treatment thus favoring angiogenesis.

IL-1b is a potent immunoregulatory and proinflammatory cytokine secreted by a variety of activated immune cells. Several studies provide evidence that angiogenesis and VEGF is IL-1 dependent [45-48] and the major transcriptional activator of the VEGF gene is HIF (Hypoxia Inducible Factor) [49]. IL-1b induced a pattern of gene expression to favor vascular permeability involving the HIF-VEGF axis [50]. Activation of the ERK1/2 pathway by IL-1b may result in the accumulation of HIF-1a protein, which initiates VEGF secretion in normal human cytotrophoblast cells. In the previous study, we observed that VEGF, HIF-1a and HIF-2a mRNA expressions in caruncle and uterus were greater in ewes supplemented with gT compared to the ewes in the CON group which is coincided with improved angiogenesis in the placental vascular network in gT group [10]. In the present study, IL-1b mRNA abundance was suppressed in gT treated ewes compared to ewes in the CON group. It is plausible that the IL-1b mRNA abundance is lower during late gestation. Studies on immunohistochemical localization of IL-1a and IL-1b in normal human placenta showed that both IL-1a and IL-1b forms are localized to villous syncytiotrophoblast and to extravillous trophoblast [51] and there is a gradual decrease of IL-1 reactivity with increasing gestational age. Also, IL-1a contributes to angiogenesis in hypoxia, when IL-1b secretion is reduced to suboptimal levels [51]. In addition, Molvarec et al., (2011) did not find association between serum levels of leptin and pro-inflammatory cytokines in preeclampsia, which might be explained--at least partly--by the fact that the latter (especially TNF-α and IL-1β) have a very short-half life in the maternal circulation [52]. In dogs, the expression was absent in uterus during diestrus (10-12 days post ovulation), and preimplantation (10-12 dpb) and placentation sites (day 20-35 dpb) [53].

The aT exerts an inhibitory effect on the release of the proinflammatory cytokine, IL-1b, via inhibition of the 5-lipoxygenase pathway [54] and aT supplementation significantly lowered levels of C-reactive protein and interleukin-6 [55]. Also, aT inhibits of IL-8 synthesis from endothelial cells [56]. It is evident that findings from these studies showed that supplementation of aT suppressed the expression of interleukins. In this study, the supplementation of aT suppressed mRNA expression of IL-1b, IL-6 and KDR in placentome, and IL-8 in caruncle compared to placebo treated ewes.

It should be noted that the protein expressions work was not performed in this study. However, protein expressions of proinflammatory cytokines and angiogenic markers in sheep placental tissues were reported in mid and late gestation [57,58].

## Conclusions

Gamma tocopherol supplementation increased IL-6, IL-8 and KDR mRNA expression, and suppressed sFlt1 mRNA expression in uterus, caruncle and cotyledon during late gestation. In addition, the VEGF mRNA expression in uterus, caruncle and cotyledon is also increased following supplementation during late gestation. The increase in VEGF mRNA abundance is enhanced via both VEGFR1 (low sFlt1) and VEGFR2 (high KDR) mRNA abundances. Taken findings from this study together, it is conceivable that the angiogenic effect of gamma tocopherol in placental vascular network is exerted via an alternate path by enhancing IL-6 and IL-8 mRNA.

## Competing interests

The authors declare that they have no competing interests.

## Authors' contributions

RK did the work of acquisition of funding, conception, design and collection of data; data analysis and interpretation, drafting of the manuscript, tables and figures. VK contributed to the design and conception, PCR analysis, collection of data and drafted the manuscript. GH and AT helped with concepts and drafting the manuscript. All authors have read and approved the final manuscript.

## Supplementary Material

Additional file 1 **Figure S1**. Photograph of the ethidium bromide-stained electrophoresis gel, with amplicons of the expected sizes. Click here for file
