# Supplementary material for: Effect of tocopherol supplementation during last trimester of pregnancy on mRNA abundances of interleukins and angiogenesis in ovine placenta and uterus
Source: Reprod Biol Endocrinol. 2012 Jan 23;10:4. doi: 10.1186/1477-7827-10-4 (PMC3398327; doi:10.1186/1477-7827-10-4)
Supplement: Additional file 1 — Figure S1. Photograph of the ethidium bromide-stained electrophoresis gel, with amplicons of the expected sizes. [file 1477-7827-10-4-S1.DOCX]

Figure S1: Photograph of the ethidium bromide-stained electrophoresis gel, with amplicons of the expected sizes.

Lad – Ladder;

Unknown – Unknown samples;

Neg- Negative control;
